# A Qualitative Study Exploring Facilitators for Improved Health Behaviors and Health Behavior Programs: Mental Health Service Users' Perspectives

**DOI:** 10.1155/2014/870497

**Published:** 2014-05-06

**Authors:** Candida Graham, Crystal Rollings, Sarah de Leeuw, Lesley Anderson, Brenda Griffiths, Nansi Long

**Affiliations:** ^1^Northern Medical Program, Department of Psychiatry, University of Northern British Columbia, 3333 University Way, Prince George, BC, Canada V2K 4C2; ^2^University of British Columbia, Vancouver, BC, Canada V6T 1Z3; ^3^School of Population and Public Health, University of British Columbia, Vancouver, BC, Canada V6T 1Z4; ^4^Activity Centre, BC Schizophrenia Society, Prince George Branch, Prince George, BC, Canada V2L 5G5

## Abstract

*Objective.* Mental health service users experience high rates of cardiometabolic disorders and have a 20–25% shorter life expectancy than the general population from such disorders. Clinician-led health behavior programs have shown moderate improvements, for mental health service users, in managing aspects of cardiometabolic disorders. This study sought to potentially enhance health initiatives by exploring (1) facilitators that help mental health service users engage in better health behaviors and (2) the types of health programs mental health service users want to develop. *Methods.* A qualitative study utilizing focus groups was conducted with 37 mental health service users attending a psychosocial rehabilitation center, in Northern British Columbia, Canada. *Results.* Four major facilitator themes were identified: (1) factors of empowerment, self-value, and personal growth; (2) the need for social support; (3) pragmatic aspects of motivation and planning; and (4) access. Participants believed that engaging with programs of physical activity, nutrition, creativity, and illness support would motivate them to live more healthily. *Conclusions and Implications for Practice.* Being able to contribute to health behavior programs, feeling valued and able to experience personal growth are vital factors to engage mental health service users in health programs. Clinicians and health care policy makers need to account for these considerations to improve success of health improvement initiatives for this population.

## 1. Introduction

Concern about the physical health of mental health service users is growing globally. Mental health service users, who experience enduring mental illness, have markedly elevated rates of cardiometabolic disturbance including obesity, diabetes, dyslipidemia, and cardiovascular disease [1–3] resulting in a disproportionate burden of health for this population and a profound effect on healthcare budgets [4]. In 2003, it was estimated that nearly 190 million people worldwide have diabetes and that the prevalence of type 2 diabetes in individuals [5] with schizophrenia can be 2–4 times higher than the general population with impaired glucose tolerance being as high as 30% [6]. Rates of diabetes are similarly high for individuals with depression; they experience an approximately 60% increased risk of developing type 2 diabetes [7]. Looking at obesity, a leading cause of preventable death in the US and other developed nations, elevated rates are observed in individuals with lifetime mood disorders [8] and schizophrenia [9]: rates of obesity in these populations are 41–50% in comparison to 27% of controls [10]. Subsequently individuals with schizophrenia or bipolar disorder have a 20–25% shorter life expectancy than the general population [11] and die at least 10 years earlier than age-matched individuals from causes other than suicide [12]. Authors point to poor health behaviors, poor engagement with health services, smoking, sedentary lifestyle, and poor diet as well as medication side effects [13–15], to account for the increased rates of cardiometabolic disease with this population. Many authors advocate adoption of a healthy lifestyle by this population, one consisting of good dietary and physical exercise habits. However, programs of physical activity, wellness training, and targeted behavioral interventions have shown only moderate improvements for mental health service users [16–22] and attrition from such programs is much higher than that for study controls [18, 20] as well as greater than exercise cessation in the general population [23]. With poor engagement by mental health service users and such high attrition rates from targeted health programs, the modest effects of such programs become further limited as to their usefulness within the community population of mental health service users. Barriers to engagement and continuance with such programs have been explored [24–28] and researchers have identified that symptoms due to illness, treatment side effects, stigma (structural, social, and self), receiving little support, and difficulties changing habits all act as significant barriers to healthier lifestyle choices for mental health service users. Authors have also, however, highlighted the need to explore facilitators and factors that might aid individuals and program development to potentiate health behavior change [25, 29].

This study therefore sought to explore two important questions, focused on improving the effectiveness of health behavior programs and health behaviors for mental health service users: (1) the types of health programs mental health service users want to develop and (2) factors that help mental health service users live healthily and engage with health programs.

## 2. Materials and Methods

We sought to understand facilitators for better health behaviors by investigating the lived experiences of the service users. A qualitative methodology was thus chosen. We were interested in thick and rich description as well as concept analysis and theory generation. As there was no preexisting notion to test and it was hoped that a theory may emerge from systematically collected data, grounded theory offered the most appropriate method [30].

The study was undertaken in a rural town in Northern British Columbia, Canada, population 80,000. Canada's healthcare needs are primarily met by a publically funded national healthcare system. A collaborative relationship previously existed between researchers from the University of Northern British Columbia and the participating psychosocial rehabilitation centre, run by the BC Schizophrenia Society [Prince George Branch]. Service users at the centre have specialist services involved with their care and are living with mental illness. The centre provides services for approximately 70 clients daily, with about 170 clients in total signed up to the centre.

Focus groups were the initial stages of a community participatory research project. The focus groups directly engaged a community of mental health service users to help empower the community and individuals to develop health improvement initiatives. These focus groups were developed following on from initial research looking at service users perspectives of and barriers to healthy living [25].

### 2.1. Procedure and Measures

Researchers undertook recruitment by presenting the proposed project to centre users and by providing the recruitment literature at the centre. Interested individuals then approached the centre coordinator and gave their details. Researchers undertook purposeful sampling/intentional sampling to identify participants who met the study criteria of (1) living with enduring mental illness, (2) being over the age of 19, (3) experiencing a stable mental state at the time of the study, (4) receiving community specialist services, and (5) being able to give informed consent. One participant was excluded as he was under the age of 19 at the time of the study.

Ethical approval for the study was gained from the relevant research ethics committees. Prior to participation, each participant gave informed written consent. Participants were compensated $50 for their time. Demographic and illness data was collected after the focus group.

In conducting the focus group discussions, as with all reconstructive methods, the basic methodological principle of allowing the groups to gain their “own structural identity” was followed [31]. This gave the discourse the opportunity of focusing on those experiences representing the focused experiential basis for the group's collective experience. Only in the later stages of the focus group did the facilitators guide and return participants to themes that had not organically arisen in discussion. The broad opening question of “we would like to know what works for you personally to live healthily” was addressed to the participants in the focus groups. The questions “how do you motivate yourself?”, “What external factors and things in the local community help you to live healthily?”, and “think about a time in the past when you successfully changed your health behavior, what helped you make those changes?” were used to facilitate the opening question if required. Once this portion of the discussion had been completed, the next topic was initiated with the broad request “we are interested to hear about healthy lifestyle projects you would like to move forward with as a community.” The focus groups were facilitated by one of two experienced researchers (Crystal Rollings and Lesley Anderson) who had previous experience facilitating focus groups but were not healthcare workers hence avoiding bias. Facilitators ensured a psychologically safe environment by highlighting the rules of confidentiality and respect prior to the interview. Using a room at the psychosocial rehabilitation centre, in which participants identified they felt comfortable, ensured safety of the physical environment. Facilitators used informal and encouraging personal styles; they allowed the group to follow its own themes occasionally using nondirective questions to clarify understanding. Facilitators observed group dynamics and encouraged participation by all members. The focus groups lasted 60 minutes.

### 2.2. Analysis

The focus groups were audiotaped and transcribed verbatim. Analysis of the transcripts was an iterative process undertaken by two independent researchers (Candida Graham and Brenda Griffiths). These researchers read and reread the text undertaking a close reading of the narratives, being careful not to move too quickly to structure the data. This allowed greater immersion into the data and decreased the likelihood of researcher bias. Thematic analysis was used to examine the data: identifying coding and organization of themes with extracts of text serving as units of analysis. The two researchers then took the position of critical peer reviewers, comparing thematic analysis and critiquing emerging themes. Discrepant themes were discussed and resolved by consensus. Any orientation frameworks developed empirically were verified by comparison with the other focus groups (i.e., how was the topic dealt with in the other groups?). NVivo 10 [32] was used to systematize themes with coded sections of transposed script. Saturation of themes occurred after the third focus group. Results were shared with the participants at informal presentations as a verification process. Participants and members of the centre identified the themes resonated with them typified by the comment that participants identified they felt “listened to.”

## 3. Results and Discussion

Thirty-seven participants who met the study criteria were recruited (19 females 18 males). All participants wanting to participate in the focus groups were accommodated to allow them a voice within the community participatory research project. The five focus groups had between 5 and 8 participants, which meets the ideal size for focus groups [33] and accommodated the availability of both participants and researchers.

The age range of participants was 26 to 72 years (*M* = 42.3 years). Twenty-one participants lived in rented accommodation, 2 owned their own home, 2 owned their own trailer, 11 lived with family, and 1 lived with a caregiver. Participant diagnoses are shown in Table 1.

### 3.1. Facilitators of a Healthy Lifestyle

When considering “facilitators of a healthy lifestyle” after an iterative process, four major themes were agreed upon: (a) factors of empowerment, self-value, and personal growth; (b) the need for social context and support; (c) pragmatic aspects of motivation and planning; and (d) access (see Table 2).

#### 3.1.1. Factors of Empowerment, Self-Value, and Personal Growth

Participants most frequently mentioned being able to experience personal value and growth, self-worth, and the ability to contribute as positive facilitators to any successful healthy lifestyle change.

Teaching, or assisting others, was identified as one of the reasons for taking part in healthy lifestyle activities. Participants were motivated to begin and continue healthy lifestyle activities if they felt their actions were assisting others. For instance, participants said, “knowing that I can make a difference in somebody else's life is important…for me that is motivational,” “I think it's an important point that you need to have a feeling that you're contributing,” and another noted, “if I'm facilitating it, I find that motivates me because I'm helping other people.”

Regarding self-discovery, participants highlighted the need for health initiatives not only to “give them room” to try different things, but also to fail and try again. “Being able to jump in, fail, succeed, discover” was, as one participant put it, very important. Another participant identified the importance of initiatives offering “something by which you can change and grow, some programs, it's the same every week, which is not good.”

The need for health initiatives to go beyond empowering the individual to empowering the mental health service user's community was also mentioned. One participant noted, “there's nothing worse than being educated by somebody that's learned stuff through a book…I do not want to be babied…but be taught by people who have been through it,” and another participant who offered his thoughts stated, “we do not need the mental health organization imposing their will on (us), as if peers cannot make up their own mind on what they want to do.”

#### 3.1.2. A Social Context and Support

The need for a social context to any health behavior initiative was the next most referenced facilitator. Specifically, this meant undertaking activities either as a group or in a buddy system. Participants asserted that a social context makes behavior change programs more fun and enjoyable thus facilitating success; “socializing, I think, I just love socializing with everyone and surrounding yourself with friends and peers that helps.” Put in another way, a participant noted, “some form of exercise certainly helps if you have a partner or a few partners, somebody to support you.” However, it was highlighted that if groups are not matched for ability, a decrease in motivation can occur. One participant summarized a previous experience related to this: “we used to do quite a bit of walking around the (local sports arena) and there was somebody that could do 18 laps and it used to kinda embarrass (those that couldn't) and they gave up.”

Having the partnership of peers undertaking behavior change activities also resulted in a sense of accountability to the buddy or group further motivating change. As expressed by one participant, “accountability to somebody else that you're doing these things that you've decided to do” and by another participant as “I think being part of a group is motivational for me, you're more accountable that way.”

An additional facilitating factor to group participation was safety. For instance one participant said, “walking in a group, you have the support and strength from your peers rather than being intimidated or anxious with strangers on the street. That coward mentality of finding an isolated, scared person, head hanging down, that's a mark (for them) but those cowards aren't going to intimidate a group.”

#### 3.1.3. Pragmatic Aspects of Motivation and Planning

Pragmatic aspects around identifying reasons for change, goals, and scheduling were the third most referenced factors facilitating change identified by participants.

Reasons for change were centered on the negative consequences of not effecting change. As one participant put it: “I was overweight and my wife was saying “look I do not want you to have a heart attack” so that gave me the incentive to change.”

Routine was mentioned as a factor that assisted participants with following through on healthy lifestyle changes. An example of this was expressed by one participant who said, “making a schedule for myself. Having things sort of organized, because if I donot I'm a scatter-brain and I donot get anything done.”

The setting of small appropriate goals was also mentioned as important to the success of healthy lifestyle changes and crucially not feeling guilty if they are not reached. For instance, a participant said that “if you have a goal, not necessarily a big goal even getting out the door sometimes is a really big step” and another eloquently highlighted the consequence of not setting appropriate goals: “I'm one of those people that needs to see results right now, you know, I should go for a walk and come back five pounds lighter. But it doesn't work that way and that's the problem, my motivation doesn't stay with me for very long.”

#### 3.1.4. Access

Having knowledge of what was available in the community to help with healthy lifestyle change, having transportation, and having venues with easy access were highlighted as facilitators to healthy lifestyle change. During the focus group, information regarding community access was shared between participants from how to access free swim passes to transportation tips. An example of this interaction and knowledge exchange is as follows: “our drawback is transportation, we should look towards something for transportation…we've got the (local community bus service) you could call him up.”

With regard, venues with easy access participants identified the desire for programs to be run from the center. As an example, a participant said, “I would love to see exercise programs brought here…then we're not dragging people all over.”

## 4. Desired Content of Healthy Lifestyle Programs

Participants identified four categories of initiative they wished to engage with: (a) physical activity, (b) nutrition, (c) creativity, and (d) illness support. The most frequently referenced category was physical activity. Within this rubric walking, was the most frequently mentioned. Walking was followed by swimming and low impact exercise such as yoga and Pilates.

Participants mentioned a number of nutrition initiatives ranging from education on healthy eating and purchasing to a community kitchen and shopping groups. For example, participants suggested “sessions or seminar of healthy cooking and eating…and (understanding food) labeling” and “shopping groups to learn how to buy healthy food.” Other participants put forward the idea of a community kitchen, “the community kitchen, where you have a bunch of people come together and they bring their food containers and you do one set of meals and everybody takes shares home, so you can have one big cooking day, learn the cooking skills for the things you haven't done, there's their meals for weeks.”

The desire to participate in “creative initiatives” was unexpectedly mentioned by participants. This was mentioned across four of the focus groups. It was expressed by one participant as “being creative, whether it's poetry, music or art or storytelling, those are all activities that fulfill a person's inner being with purpose and if you're in a group with common interests in a supportive manner more and more flows out of that” and expressed by another as “one of the things that I do to keep myself healthy is different ways of expressing my feelings that might be non-verbal, like art, music and things like that.” One participant powerfully illustrated the benefit creativity had for them: “I suffered from alcoholism for years and then I started drawing, like I just drew a moon and stars and started from there.”

Participants in four focus groups mentioned the desire to develop illness support and education programs, for example, “(the schizophrenia society) workshops gave me knowledge of what was happening to me, I had mental illness and I didn't even know what that was. I thought it was being retarded…the more you know what's going on with you, that's a big part of my staying healthy.”

Few studies have specifically explored mental health service users perspectives of incentives to participation in lifestyle interventions but authors reviewing a number of studies [29] highlighted that encouragement from others, buddy and group activities, choice and variety, and rewards for participation and self-efficacy may be positive incentives to participation in healthy lifestyles. Service providers have identified their beliefs that clients experience positive facilitation, in living more healthily, when, taking personal initiative, having peer support and easy access to community resources [34].

This study finds four incentives, or facilitators, that mental health service users identify as helping them to make healthy behavior changes in their lives: (a) factors of empowerment, self-value, and personal growth; (b) the need for social context and support; (c) pragmatic aspects such as reasons for change, goal setting, and scheduling; and (d) access. Additionally the original participatory methods of this study are innovative, thus adding and expanding knowledge in the field.

The need for stakeholder involvement when planning public health interventions has been identified, by a review of the grey literature [35], and authors postulate that self-determination and self-efficacy are important factors to engage mental health service users in meaningful health behavior change and treatment adherence [36]. However, we believe this is the first study in which mental health service users have directly identified factors of empowerment, self-value, and personal growth as incentives or facilitators to improved health behavior change. This can be observed as an expressed tendency towards self-determination [36] and self-actualization [37, 38] by mental health service users in the endeavors they choose to participate in, an aspect not frequently recognized by mental health service providers.

Social support has been reported as contributing to observed benefits of health interventions for mental health service users in studies [27, 29, 39] and this study supports these findings with the importance of social support identified as (1) creating a supportive, fun environment; (2) creation of accountability for the individual to the group; and (3) safety.

Participants highlighted pragmatic factors such as identifying reasons for change, setting small achievable goals, and scheduling activities as being positive facilitators to change. These facilitating factors have been identified by authors in the context of motivational interviewing as “helping people change” [40], validating mental health service users insights into what helps them to improve their health behaviors.

Finally, having knowledge and transportation to allow access to opportunities in the local community was identified as important facilitators that could enable mental health service users to improve their health behaviors. Previous authors have observed transportation problems causing nonattendance at an organized health improvement initiative [41] and service providers have highlighted transport to community resources facilitating healthy behaviors [34]. The factors of knowledge and transport equating to “access” for mental health service users may be obvious but we believe have not been highlighted in this way in the literature to date.

A previous survey identified walking as the most popular physical activity for mental health service users [28] and other authors have highlighted walking being part of a regular routine for more active mental health service users [26]. This is supported by this study in that participants identified walking as the most referenced preferred physical activity; however, this study asked the broader question “what healthy lifestyle projects would you like to move forward with as a community?” which yielded the expected results of physical activity and nutrition initiatives and also the completely unexpected theme of a creativity initiative. Participants identified that this would allow self-reflection, self-expression, personal growth, and development as individuals, thereby leading them to make healthier lifestyle choices, as was powerfully illustrated by the participant who had alcohol problems, and starting to draw was the start of their rehabilitation.

### 4.1. Limitations

Potential limitations of the study are the selection bias with individuals self-selecting for the study and the participants attending a psychosocial rehabilitation centre. Generalizability of the results is limited by the fact that participants are from one health authority, in Northern British Columbia, and were stable with regard to their mental illness. The use of economic incentives for research participation may result in participants being motivated by compensation rather than wanting to contribute to the project; however, the researchers had a prior relationship with the community and these focus groups were part of a larger community participatory research project aiming to empower the community. This resulted in motivated participants providing rich material within the focus groups and many participants had to be reminded of the compensation after participation in the groups. Despite these limitations, the study adds depth by providing a rural perspective to research undertaken with more urban populations and the rich data enlightens and informs those working in the field.

## 5. Conclusions and Clinical Recommendations

When considering facilitators or incentives to health behavior change, mental health service users identify four major themes: (a) factors of empowerment, self-value, and personal growth; (b) the need for social support; (c) pragmatic/motivational interviewing aspects such as reasons for change, goal setting, and scheduling; and (d) access. These factors are required if health initiatives and health improvement programs are to be successful in engaging mental health service users with health behavior change. Additionally, while physical activity and nutrition programs have shown effectiveness in improving health behaviors studies, looking at the effectiveness of creative initiatives against these should be considered.

## Figures and Tables

**Table 1:** Participants' living arrangements and diagnoses.

Variable	*N* = 37
	*n* (% of sample)
Living arrangements	
Rented accommodations	21 (56.76%)
Own home	2 (5.41%)
Own trailer	2 (5.41%)
Live with family	11 (29.73%)
Live with caregiver	1 (2.70%)
Diagnoses*	
Neurodevelopmental disorders	5 (13.51%)
Schizophrenia spectrum disorders	9 (24.32%)
Bipolar and related disorders	12 (32.43%)
Depressive disorders	13 (35.14%)
Anxiety disorders	7 (20.00%)
Obsessive-compulsive disorders	1 (2.70%)
Trauma and stress related disorders	3 (8.11%)
Substance and addictive disorders	4 (10.81%)
Neurocognitive disorder	1 (2.70%)

*Note: 17 participants had disorder comorbidity.

**Table 2:** Participants' identified facilitators of healthy lifestyles.

Theme	Exemplary quote(s)
Facilitators	
Empowerment, self-value, and personal growth	“Knowing that I can make a difference in somebody else's life is important…for me that is motivational,” “Being able to jump in, fail, succeed, discover,” “something by which you can change and grow, some programs, it's the same every week, which isn't good,”
Social support	“Some form of exercise certainly helps if you have a partner or a few partners, somebody to support you,” “I think being part of a group is motivational for me, you're more accountable that way,” “walking in a group, you have the support and strength from your peers rather than being intimidated or anxious with strangers on the street. That coward mentality of finding an isolated, scared person, head hanging down, that's a mark (for them) but those cowards aren't going to intimidate a group,”
Motivation and planning	“Making a schedule for myself. Having things sort of organized, because if I don't I'm a scatter-brain and I don't get anything done,” “if you have a goal, not necessarily a big goal even getting out the door sometimes is a really big step,”
Access	“I would love to see exercise programs brought here…then we're not dragging people all over.”
